# Clinical Validation of Explainable Deep Learning Model for Predicting the Mortality of In-Hospital Cardiac Arrest Using Diagnosis Codes of Electronic Health Records

**DOI:** 10.31083/j.rcm2409265

**Published:** 2023-09-21

**Authors:** Chien-Yu Chi, Hadi Moghadas-Dastjerdi, Adrian Winkler, Shuang Ao, Yen-Pin Chen, Liang-Wei Wang, Pei-I Su, Wei-Shu Lin, Min-Shan Tsai, Chien-Hua Huang

**Affiliations:** ^1^Department of Emergency Medicine, National Taiwan University Hospital Yunlin Branch, 640 Yunlin, Taiwan; ^2^Knowtions Research Inc., Toronto, Ontario M5J 2S1, Canada; ^3^Department of Emergency Medicine, National Taiwan University, 100 Taipei, Taiwan

**Keywords:** in-hospital cardiac arrest, artificial intelligence, explainable deep learning model, SHAP, electronic health record

## Abstract

**Background:**

Using deep learning for disease outcome prediction is an approach that has made large advances in recent years. Notwithstanding its excellent performance, clinicians are also interested in learning how input affects prediction. Clinical validation of explainable deep learning models is also as yet unexplored. This study aims to evaluate the performance of Deep SHapley Additive exPlanations (D-SHAP) model in accurately identifying the diagnosis code associated with the highest mortality risk.

**Methods:**

Incidences of at least one in-hospital cardiac arrest (IHCA) for 168,693 patients as well as 1,569,478 clinical records were extracted from Taiwan’s National Health Insurance Research Database. We propose a D-SHAP model to provide insights into deep learning model predictions. We trained a deep learning model to predict the 30-day mortality likelihoods of IHCA patients and used D-SHAP to see how the diagnosis codes affected the model’s predictions. Physicians were asked to annotate a cardiac arrest dataset and provide expert opinions, which we used to validate our proposed method. A 1-to-4-point annotation of each record (current decision) along with four previous records (historical decision) was used to validate the current and historical D-SHAP values.

**Results:**

A subset consisting of 402 patients with at least one cardiac arrest record was randomly selected from the IHCA cohort. The median age was 72 years, with mean and standard deviation of 69 ± 17 years. Results indicated that D-SHAP can identify the cause of mortality based on the diagnosis codes. The top five most important diagnosis codes, namely respiratory failure, sepsis, pneumonia, shock, and acute kidney injury were consistent with the physician’s opinion. Some diagnoses, such as urinary tract infection, showed a discrepancy between D-SHAP and clinical judgment due to the lower frequency of the disease and its occurrence in combination with other comorbidities.

**Conclusions:**

The D-SHAP framework was found to be an effective tool to explain deep neural networks and identify most of the important diagnoses for predicting patients’ 30-day mortality. However, physicians should always carefully consider the structure of the original database and underlying pathophysiology.

## 1. Introduction

The incidence of in-hospital cardiac arrest (IHCA) is about 8.5 records for every 1000 admissions [1]. For IHCA patients, the rate of survival to hospital discharge is about 39.5%, and only 28.3% of IHCA patients regain independent or partially independent lives [2]. Previous studies suggest that detecting adverse signs and symptoms early and adjusting medical care accordingly has the potential to improve a patient’s prognosis by properly allocating healthcare resources and reducing future healthcare needs [3]. Machine learning methods, especially deep learning approaches, have been shown to be more effective than traditional epidemiological studies at uncovering disease patterns and understanding patient disease trajectories [4, 5, 6, 7, 8]. However, since prior selection of potential risk factors is required in epidemiological research methods, these approaches are time consuming and prone to bias if conducted manually. Although machine-learning approaches provide promising levels of prediction accuracy, their lack of interpretability has limited their adoption in a clinical setting. It is important to develop a robust and trustworthy framework consisting of interpretable methods that can explain why a certain prediction was made for a given case [9, 10, 11]. However, there are relatively few studies regarding explainable deep learning models for cardiac arrest prediction. Some researchers also advocate for a careful and thorough validation of these approaches [12], which has not yet been undertaken.

In this study, we used a pre-trained Hierarchical Vectorizer (HVec) deep learning model to predict the mortality of cardiac arrest patients using data from Taiwan’s large-scale National Health Insurance Research Database (NHIRD). This model achieved a 0.711 area under the receiver operating characteristic (AUROC) score when predicting patients’ 30-day mortality after each clinical record and a 0.808 AUROC score when predicting patients’ 30-day mortality after IHCA [13]. Based on this, we introduced a deep learning interpretation Deep SHapley Additive exPlanations (D-SHAP) framework to determine the correlation between input features and 30-day mortality probability of IHCA patients. In clinical settings, the diagnosis code is a key feature used by physicians to estimate patient health status. The diagnosis code input feature was used to check the performance of the D-SHAP framework. A linear combination method is proposed to aggregate the SHAP values and thus generate the impact of the diagnosis code on the mortality probability [14, 15]. The physicians’ opinion was introduced as the benchmark to measure the similarity between the impact calculated from the D-SHAP framework and human experts’ analyses.

In this study, we aim to evaluate that D-SHAP can capture the diagnosis code with the highest mortality risk from a deep neural network and generate a result consistent with the physician’s diagnosis.

## 2. Methods

This study was approved by the Institutional Review Board of National Taiwan University Medical College.

### 2.1 NHIRD Dataset

Taiwan’s NHIRD is one of the most comprehensive data sources among all national electronic health record (EHR) databases around the world. It is a huge database that includes up to 99.99% of Taiwan’s population [16]. NHIRD is intended for reimbursement purposes, and claim data includes patients’ medical information such as gender, age, date of inpatient or outpatient visits, medication, procedures, discharge status, and the total health cost of each visit. Details of patients’ medical history and bedside information, including laboratory test results, vital signs, and physical examination, are not recorded in the NHIRD.

### 2.2 Cardiac Arrest Dataset

In this study, a sufficiently large subsample of this database has been utilized to train, test, validate, and interpret our model. Patients who had at least one IHCA event during the study period (from January 1, 2002 to December 31, 2010) were included in the analysis. International Classification of Disease, 9th Revision (ICD-9) was used in the dataset. The following ICD-9 codes have been used in this study for identifying the ICHA cohort: procedure codes 99.60 (cardiopulmonary resuscitation, not otherwise specified) and 99.63 (closed-chest cardiac massage) [17]. Extract, Transform, & Load (ETL) was performed on the raw dataset to prepare an organized database. To improve the raw data organizationally, the database was regrouped into three major categories: insurer, person, and caregiver. Meanwhile, vocabulary tables were constructed based on extracted concepts used in the raw data [13].

The resulting database consists of 4,622,079 clinical records, both inpatient and outpatient, from 168,693 people (mean and standard deviation of records per person 9.30 ± 10.90) who have experienced at least one IHCA over a nine-year period. A total of 3,052,601 dental, traditional medicine, and local clinic records were excluded from the analysis. These records were usually repetitive and not particularly relevant to critical illness, so they would simply have added noise to the machine learning models. The remaining 1,569,478 clinical records were included in the final analysis.

### 2.3 Deep SHapley Additive exPlanations (D-SHAP) Deep Learning Models

Deep SHapley Additive exPlanations (D-SHAP) is a method that can provide deep learning model explanation using linear approximation and derivative chain rule for each input and output, referred to as local input/output [17]. The methodology and calculation details were provided in the **Supplementary Material** for reference and further examination. The explainable deep learning model will provide each clinical record a continuous D-SHAP value for predicting the probability of 30-day mortality.

**Fig. 1.**
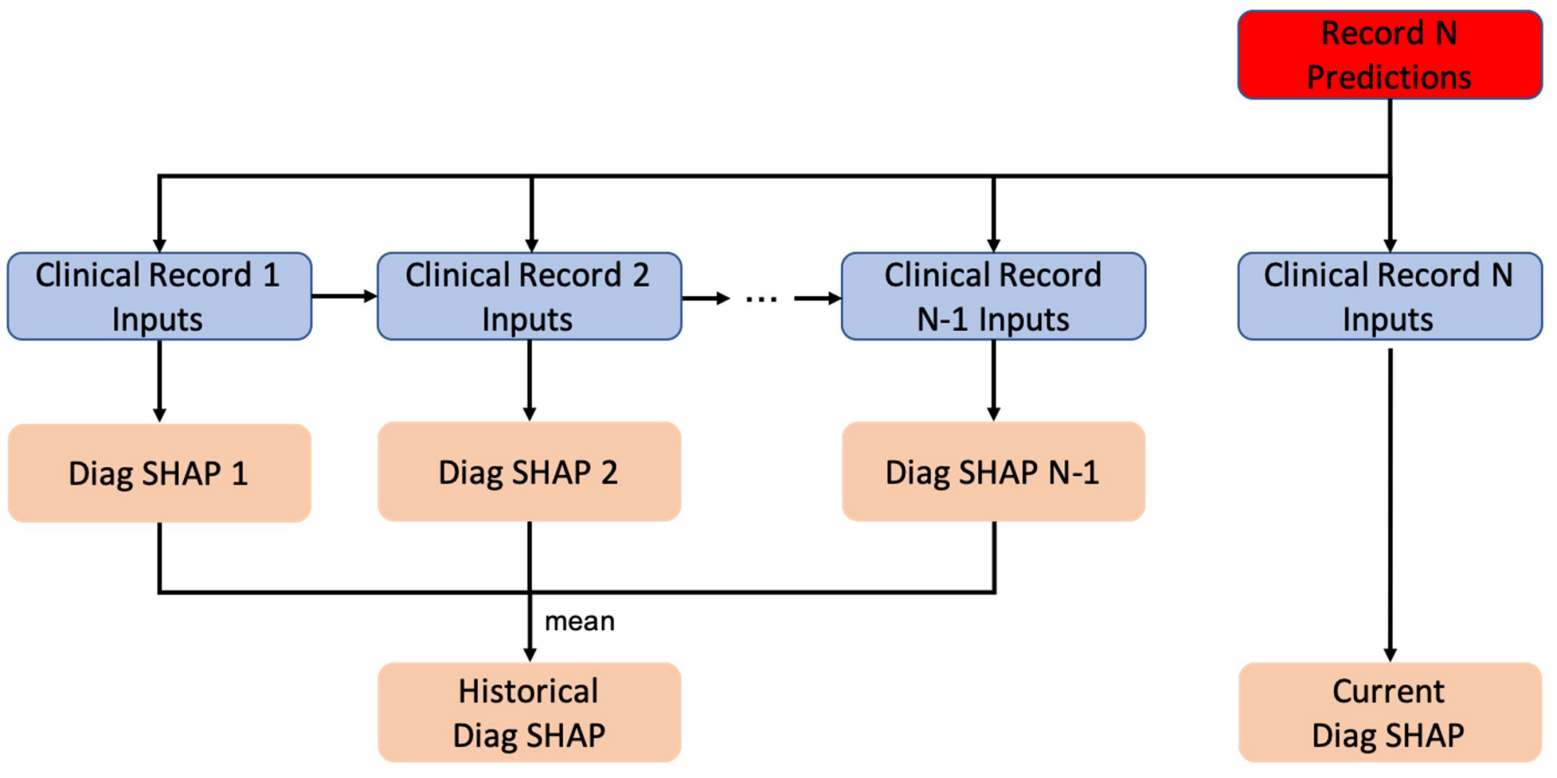
**Aggregating historical diagnosis SHapley Additive exPlanations (SHAP) versus current diagnosis SHAP**.

For the diagnosis codes in each record, current and historical D-SHAP impacts are analyzed from the model’s perspective to determine the importance of each diagnosis code (Fig. 1). The current D-SHAP value is defined by checking the diagnosis codes of the current event, providing a scale to measure the likelihood of 30-day mortality for the individual. The historical D-SHAP value is defined by checking the diagnosis codes of all previous events and provides a scale to measure the likelihood of 30-day mortality. In this experiment, the high/low SHAP impact segmentation criterion is set according to the results. In principle, records with SHAP prediction value ≥0.25 are considered as high SHAP impact, and these diagnosis codes can predict 30-day mortality. In contrast, records with SHAP prediction value ≤0.10 are considered as low SHAP impact; these diagnosis codes can negatively predict 30-day mortality.

In this study, the SHAP value is validated against a physician’s decision to determine the consistency between the SHAP value and human knowledge.

### 2.4 Validation Dataset by Clinical Judgment

A subset of 402 patients with IHCA records randomly selected from 168,693 people in our NHIRD dataset was used to compare differences among D-SHAPE models as well as decisions made by human physicians. The physicians’ opinions served as the reference point for assessing the congruity between the D-SHAP framework and human experts’ analyses. For each patient, the IHCA records with 30-day mortality and four consecutive historical records prior to that record were used for analysis. Each visit was given a current decision point and a historical decision point. Physicians were asked to provide opinions and assign a scale of 1-to-4-point denoting the possibilities of 30-day mortality (1 denotes high probability of 30-day mortality, 2 denotes medium probability, 3 denotes low probability, and 4 denotes very low probability).

In correspondence with the SHAP algorithm (Fig. 1), current decision points were denoted after physicians judged all diagnoses within an individual visit. Historical decision points were denoted for each visit by considering the diagnosis of that visit and the previous four records together. Therefore, each patient would be designated with 5 current decision points and 4 historical decision points. D-SHAP impact values of diagnosis code greater than 0.25 are considered to indicate high-impact records corresponding to scale 1 (high probability of 30-day mortality) in clinical judgment by physicians. D-SHAP impact values of diagnosis code less than 0.10 are considered to indicate low-impact records corresponding to scale 4 (very low probability of 30-day mortality).

In total, eight physicians from National Taiwan University Hospital participated in this study, and each visit was evaluated by two physicians. If the difference in annotations by the two physicians was greater than 1, the final decision was made by the authors (CYC and CHH). The physicians were blind to the model performance and patients’ outcomes when submitting their judgments.

### 2.5 Comparison of D-SHAP Model with Clinical Judgment

In order to match the clinical judgment against the D-SHAP model and avoid the misleading of some rare diagnoses, some statistics for the diagnosis codes are determined as follows:

∙**Count-high**: total counts of the diagnosis codes appear in scale 1 clinical records.

∙**Count-low**: total counts of the diagnosis codes appear in scale 4 clinical records.

∙**High-ratio**: count-high divided by the total records with scale 1.

∙**Low-ratio**: count-low divided by the total records with scale 4.

Finally, we assign each diagnosis code an importance value to describe its severity in terms of its relationship to mortality:

∙**Importance**: the difference between high-ratio and low-ratio.

The importance ranking of each diagnosis according to physicians’ opinion was set as benchmark. This benchmark was then compared with the importance ranking generated by the D-SHAP framework.

## 3. Results

### 3.1 Baseline Characteristics of IHCA Cohort

**Fig. 2.**
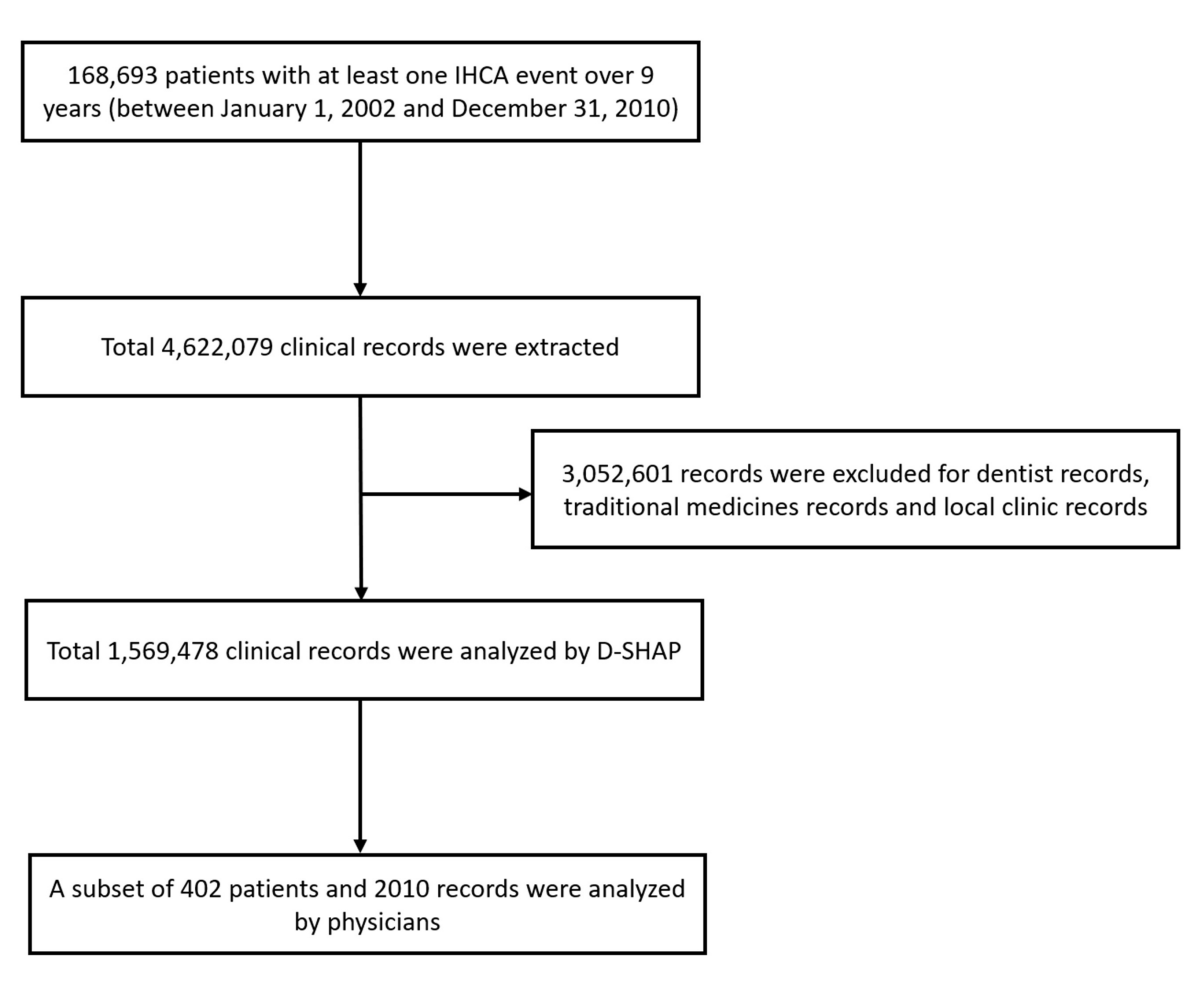
**CONSORT diagram of the study cohort and the validation data set**. IHCA, in-hospital cardiac arrest; D-SHAP, Deep SHapley Additive exPlanations.

CONSORT diagram of the study cohort and the validation data set was illustrated in Fig. 2. Among these 1,569,478 clinical records, there are 173,345 IHCA records (11.04% of the total); on average, each subject in the IHCA cohort has 1.02 IHCA records. The age of individuals in the dataset ranges from 0 to 118 years (mean and standard deviation 68.66 ± 18.96 years), including 104,691 females and 64,002 males. 164,322 subjects (97.4%) have had cardiac arrest only once, 4174 (2.4%) have had cardiac arrest twice, and only 197 (0.2%) have had cardiac arrest more than twice. We observed 87,311 death records (a 51.75% mortality rate). Of these 87,311 individuals, 82,225 passed away during their first cardiac arrest hospitalization (94.17%). For validation and interpretation of model performance, a set of physician’s annotations are needed. To this end, a subsample of the final dataset was annotated by physicians and utilized in validation and interpretation.

### 3.2 Physician Annotation Analysis

Among the subset of 402 patients in the validation dataset, the median age was 72 years with mean and standard deviation of 69 ± 17 years. The dataset consists of more males than females (female to male ratio = 0.37). Diabetes mellitus, acute respiratory failure, and pneumonia were the most frequent diagnosis codes in these individuals’ current and historical health records, as illustrated in Table 1. In Table 2, the top ten most frequent diagnosis codes were compared for individuals with a 30-day mortality versus those without. Although diabetes mellitus was the most prevalent disease across the whole cohort, acute respiratory failure was most frequent for those who had a 30-day mortality incident in their records.

**Table 1.** **Top ten most frequent diagnosis codes within individual health records in the validation dataset**.

Diagnosis code	Frequency (% of all diagnosis records)
Diabetes mellitus	4.93
Acute respiratory failure	4.81
Pneumonia	4.54
Urinary tract infection	4.19
Sepsis	2.59
Congestive heart failure	2.52
Hypertension	2.40
Chronic renal disease	2.01
Shock, unspecified	1.50
Chronic lung disease	1.50

**Table 2.** **Comparison of the top ten most frequent diagnosis codes for individuals with and without 30-day mortality records in the validation dataset**.

Diagnosis code with 30-day mortality	Frequency (% of all diagnosis records)	Diagnosis code without 30-day mortality	Frequency (% of all diagnosis records)
Acute respiratory failure	2.73	Diabetes mellitus	4.68
Sepsis	1.85	Urinary tract infection	3.90
Pneumonia	1.85	Pneumonia	3.81
Shock, unspecified	1.40	Acute respiratory failure	3.73
Urinary tract infection	0.73	Congestive heart failure	2.30
Diabetes mellitus	0.63	Hypertension	2.29
Chronic renal disease	0.59	Sepsis	1.86
Acute kidney injury	0.59	Chronic renal failure	1.78
Congestive heart failure	0.56	Chronic lung disease	1.45
Aspiration pneumonia	0.38	Acute exacerbation of chronic obstructive lung disease	1.34

The top ten most important diagnosis codes for both current and historical decisions are shown in Tables 3,4. From these tables we can see that acute respiratory failure, pneumonia, sepsis, shock, unspecified, acute kidney injury, congestive heart failure, and aspiration pneumonia appear in both current and historical top ten important diseases.

**Table 3.** **Diagnosis code importance based on current decision by physicians; top ten most important diagnosis codes leading to 30-day mortality**.

Current decision					
Diagnosis	Count-high	Count-low	High-ratio	Low-ratio	Importance
Acute respiratory failure	199	0	59.58%	0.00%	59.58%
Pneumonia	105	29	31.44%	6.40%	25.04%
Sepsis	86	4	25.75%	0.88%	24.87%
Shock, unspecified	66	0	19.76%	0.00%	19.76%
Acute kidney injury	32	1	9.58%	0.22%	9.36%
Congestive heart failure	37	14	11.08%	3.09%	7.99%
Cardiac arrest	26	0	7.78%	0.00%	7.78%
Aspiration pneumonia	21	3	6.29%	0.66%	5.63%
Cardiogenic shock	17	0	5.09%	0.00%	5.09%
Acute myocardial infarction	17	0	5.09%	0.00%	5.09%

**Table 4.** **Diagnosis code importance based on historical decisions by physicians; top ten most important diagnosis codes leading to 30-day mortality**.

Historical decision					
Diagnosis	Count-high	Count-low	High-ratio	Low-ratio	Importance
Acute respiratory failure	240	0	49.69%	0.00%	49.69%
Pneumonia	143	6	29.61%	4.44%	25.16%
Sepsis	95	1	19.67%	0.74%	18.93%
Shock, unspecified	78	0	16.15%	0.00%	16.15%
Acute kidney injury	42	0	8.70%	0.00%	8.70%
Congestive heart failure	55	5	11.39%	3.70%	7.68%
Acute exacerbation of chronic obstructive lung disease	35	0	7.25%	0.00%	7.25%
Chronic renal disease	44	4	9.11%	2.96%	6.15%
Chronic lung disease	37	3	7.66%	2.22%	5.44%
Aspiration pneumonia	26	0	5.38%	0.00%	5.38%

### 3.3 D-SHAP Analysis

To align the results with Tables 3,4, the high/low ratio and importance of each diagnosis code by current and historical D-SHAP models are presented in Tables 5,6, respectively. In Tables 5,6, the *Rank* column represents the importance of these diagnosis codes in order of physicians’ judgment as shown in Tables 3,4.

**Table 5.** **Diagnosis code importance based on current D-SHAP; top ten most important diagnosis codes leading to 30-day mortality**.

Current D-SHAP				
Diagnosis	High-ratio	Low-ratio	Importance	Rank
Acute respiratory failure	62.41%	1.14%	61.27%	1
Sepsis	36.84%	0.76%	36.08%	3
Pneumonia	42.11%	7.20%	34.91%	2
Shock, unspecified	32.33%	0.00%	32.33%	4
Acute kidney injury	14.29%	0.38%	13.91%	5
Urinary tract infection	16.54%	10.61%	5.94%	365
Hypoxic encephalopathy	5.26%	0.38%	4.88%	14
Hypertension	4.51%	1.14%	3.37%	16
Gastrointestinal bleeding	6.02%	2.65%	3.36%	12
Cardiac arrest	3.01%	0.00%	3.01%	11

D-SHAP, Deep SHapley Additive exPlanations.

**Table 6.** **Diagnosis code importance based on historical D-SHAP; top ten most important diagnosis codes leading to 30-day mortality**.

Historical D-SHAP				
Diagnosis	High-ratio	Low-ratio	Importance	Rank
Acute respiratory failure	62.50%	0.79%	61.71%	1
Sepsis	50.00%	0.79%	49.21%	3
Shock, unspecified	47.73%	0.00%	47.73%	4
Pneumonia	34.09%	8.66%	25.43%	2
Acute kidney injury	18.18%	0.26%	17.92%	5
Cardiogenic shock	9.09%	0.00%	9.09%	15
Myocardial infarction	6.82%	0.00%	6.82%	32
Cardiac arrest	6.82%	0.26%	6.56%	11
Gastrointestinal bleeding	10.23%	4.46%	5.77%	12
Aspiration pneumonia	7.95%	2.36%	5.59%	10

D-SHAP, Deep SHapley Additive exPlanations.

We see that the top-five diagnosis codes in both Tables 5,6 are consistent with the physician’s decision in Tables 3,4 despite the ordering being slightly different and the importance being more significant than the other diagnosis codes. Most of the important diagnosis codes found by D-SHAP are also considered important diagnoses by physicians. It is interesting that the diagnosis code for urinary tract infection shows up as the sixth most important diagnosis for current D-SHAP impact but only the 365th most important based on the physician’s current decision. Urinary tract infection is a common disease which is not always life threatening. However, we notice that in our dataset there are several co-prevalent comorbidities with urinary tract infection that can lead to mortality, which misleads our D-SHAP analysis process. The top-five comorbidities by prevalence in patients with urinary tract infection diagnosis included pneumonia (23.36%), diabetes mellitus (20.72%), acute respiratory failure (19.41%), sepsis (11.51%), and hypertension (8.55%).

## 4. Discussion

In this paper, we proposed a D-SHAP machine learning model that can be used to explain deep neural network modeling. The electronic health records of an IHCA cohort were investigated using our D-SHAP framework to find the most important diagnosis codes leading to mortality. After comparison with physicians’ annotations, we found that most of the important diagnosis codes that could lead to mortality can be captured by our D-SHAP framework. One of the diagnoses, urinary tract infection, showed a high discrepancy between our D-SHAP model and clinical judgment. Urinary tract infection is a relatively common disease leading to admission, especially in seniors or patients with multiple comorbidities [18]. We assume that the high prevalence of urinary tract infection in our dataset with its high frequency of comorbidities with dangerous diagnoses including pneumonia, acute respiratory failure, and sepsis might mislead the machine learning process. Results show that our framework can determine some vital diagnosis codes that cannot be found by conventional clinical judgment. However, physicians should always carefully evaluate the results of machine learning and consider underlying pathophysiological mechanisms.

Along with the recent explosive development of machine learning in medicine, several arguments about its utility in clinical practice have manifested, especially regarding black-box and overfitting issues [19, 20, 21, 22, 23]. With improvements in computer science, explainable machine learning models have been widely used recently to address the drawbacks of traditional machine learning models; they have been used in several areas of medicine [5, 24, 25, 26, 27, 28, 29], and they also provide prediction algorithms for use by clinical physicians [30]. These studies proposed several models with high predictive values for critical illness. They also proposed several predictive factors using explainable deep learning models such as SHAP and locally interpretable model-agnostic explanations (LIME), yielding insight into the mechanisms of these models. However, how reasonable these generated factors are is still in question. In addition to post-hoc judgment based on clinical rationales, which carry the risk of confirmation bias, further double-blind studies are needed for more rigorous validation [12].

In this study, not only did we propose a deep learning interpretation framework for predicting mortality by EHRs of NHIRD, but we also performed a prospective validation against the judgment of clinical physicians. To the best of our knowledge, this is the first study using a prospective method to validate an explainable deep learning model. We used diagnosis for the index as an important feature of EMRs that covers patients’ overall status as well as physicians’ judgment. To correspond with D-SHAP values, we innovated a 1–4 score for each visit by clinical judgment. In this experiment, the prevalence of each diagnosis is a key issue. Some diagnoses seldom appeared and had a very small sample size, so we cannot solely calculate the mean score of each diagnosis. Also, some diagnoses are strong predictive factors for 30-day mortality while other diagnoses are strong protective factors. Therefore, we propose measuring the importance of a diagnosis by calculating the difference in probability between high and low scores. However, the prevalence of diagnoses within the dataset was still a major confounding factor. In addition to those diagnoses with higher risk, those with higher prevalence will also have higher ranking. For example, cardiac arrest should be a stronger predictor of mortality than any other. However, due to the relatively low frequency of cardiac arrest featuring as the diagnosis, the importance of this diagnosis scored lower than other more common diseases such as pneumonia or respiratory failure. Also, frequently occurring diseases such as urinary tract infections are expected to have higher rankings, especially those with more co-prevalence with other severe comorbidities. Therefore, the ranking in our study did not emphasize the order of severity but only indicated those diagnoses that should bear greater consideration. This experiment also illustrated the point that the end users of machine learning models should always carefully evaluate the results and consider the structure of the original database.

This study has several implications. We found that an explainable deep learning model can determine diagnosis with clinical significance for a complicated database such as NHIRD. This model can be utilized as an early warning system for patients who are at risk of mortality according to recent EHRs. Patients with a high risk of mortality could be identified and re-evaluated at each clinical visit. With an explainable deep learning model, several diagnoses or risk factors can be proposed for helping physicians to make the most effective clinical decisions. We consider the present study as a preliminary study for future work and demonstrate that our model can be an effective tool with reasonable explainability. In Taiwan, the NHI database contains over 99% of the population’s medical information for insurance purposes. In the future, we hope to establish an alarm system based on NHIRD by connecting hospital EHRs and deep learning software within NHIRD, which can be universally applied to Taiwan’s population for predicting severe, high-risk medical conditions such as cardiac arrest [31, 32, 33, 34, 35].

This study had several limitations. First, the IHCA cohort was retrospectively collected using NHIRD. These patients were usually diagnosed with critical illnesses and multiple comorbidities during the study period. The implications of extending this model to the general population or other datasets are unknown. Second, only the diagnosis code was used in this study due to study design and the complexity of NHIRD. Explainability of the whole model was not evaluated or validated by this study. Third, as mentioned above, the prevalence of each diagnosis would have an impact on its calculated importance. Since NHIRD is used for reimbursement purposes, diagnoses other than primary diagnosis for admission were not always recorded by physicians. The gap between NHIRD records and clinical diagnosis should be considered. Fourth, the calculation formula for the importance of each diagnosis was designed solely for our validation experiment. Due to the lack of similar studies in the literature, the methodology used in this study should be applied with caution and further validation is needed. Finally, further studies are needed to evaluate the utility of explainable deep learning models in real-world medical applications and thus determine whether this system can improve patients’ outcomes.

## 5. Conclusions

In this study, the D-SHAP framework was found to be an effective tool for explaining deep neural networks in the prediction of patients’ 30-day mortality. Most of the important diagnosis codes that could lead to mortality, including respiratory failure, sepsis, pneumonia, shock, and acute kidney injury, can be captured by our D-SHAP framework. However, physicians should always carefully evaluate the results of machine learning, taking into account underlying pathophysiological mechanisms.

## Data Availability

The data that support the findings of this study are available from NHIRD but restrictions apply to the availability of these data, which were used under license for the current study, and so are not publicly available. Data are however available from the authors upon reasonable request and with permission of NHIRD.

## Supplementary Material

Supplementary material associated with this article can be found, in the online version, at https://doi.org/10.31083/j.rcm2409265.

## References

[1] Rasmussen TP, Riley DJ, Sarazin MV, Chan PS, Girotra S (2022). Variation Across Hospitals in In-Hospital Cardiac Arrest Incidence Among Medicare Beneficiaries. *JAMA Network Open*.

[2] Pound G, Jones D, Eastwood GM, Paul E, Hodgson CL, ANZ-CODE Investigators (2020). Survival and functional outcome at hospital discharge following in-hospital cardiac arrest (IHCA): A prospective multicentre observational study. *Resuscitation*.

[3] Hoot NR, Aronsky D (2008). Systematic review of emergency department crowding: Causes, effects, and solutions. *Annals of Emergency Medicine*.

[4] Kareemi H, Vaillancourt C, Rosenberg H, Fournier K, Yadav K (2021). Machine Learning Versus Usual Care for Diagnostic and Prognostic Prediction in the Emergency Department: A Systematic Review. *Academic Emergency Medicine*.

[5] Thorsen-Meyer HC, Nielsen AB, Nielsen AP, Kaas-Hansen BS, Toft P, Schierbeck J (2020). Dynamic and explainable machine learning prediction of mortality in patients in the intensive care unit: a retrospective study of high-frequency data in electronic patient records. *The Lancet. Digital Health*.

[6] Nielsen AB, Thorsen-Meyer HC, Belling K, Nielsen AP, Thomas CE, Chmura PJ (2019). Survival prediction in intensive-care units based on aggregation of long-term disease history and acute physiology: a retrospective study of the Danish National Patient Registry and electronic patient records. *The Lancet. Digital Health*.

[7] Meyer A, Zverinski D, Pfahringer B, Kempfert J, Kuehne T, Sündermann SH (2018). Machine learning for real-time prediction of complications in critical care: a retrospective study. *The Lancet Respiratory Medicine*.

[8] Kwon JM, Lee Y, Lee Y, Lee S, Park J (2018). An Algorithm Based on Deep Learning for Predicting In-Hospital Cardiac Arrest. *Journal of the American Heart Association*.

[9] Reddy S (2022). Explainability and artificial intelligence in medicine. *The Lancet Digital Health*.

[10] Linardatos P, Papastefanopoulos V, Kotsiantis S (2020). Explainable AI: A Review of Machine Learning Interpretability Methods. *Entropy*.

[11] Shah NH, Milstein A, Bagley SC (2019). Making Machine Learning Models Clinically Useful. *Journal of the American Medical Association*.

[12] Ghassemi M, Oakden-Rayner L, Beam AL (2021). The false hope of current approaches to explainable artificial intelligence in health care. *The Lancet Digital Health*.

[13] Chi CY, Ao S, Winkler A, Fu KC, Xu J, Ho YL (2021). Predicting the Mortality and Readmission of In-Hospital Cardiac Arrest Patients With Electronic Health Records: A Machine Learning Approach. *Journal of Medical Internet Research*.

[14] Lundberg SM, Nair B, Vavilala MS, Horibe M, Eisses MJ, Adams T (2018). Explainable machine-learning predictions for the prevention of hypoxaemia during surgery. *Nature Biomedical Engineering*.

[15] Lundberg S, Lee SI (2017). A Unified Approach to Interpreting Model Predictions. *arXiv*.

[16] Hsieh CY, Su CC, Shao SC, Sung SF, Lin SJ, Kao Yang YH (2019). Taiwan’s National Health Insurance Research Database: past and future. *Clinical Epidemiology*.

[17] DeZorzi C, Boyle B, Qazi A, Luthra K, Khera R, Chan PS (2019). Administrative Billing Codes for Identifying Patients With Cardiac Arrest. *Journal of the American College of Cardiology*.

[18] Gruneir A, Bell CM, Bronskill SE, Schull M, Anderson GM, Rochon PA (2010). Frequency and pattern of emergency department visits by long-term care residents–a population-based study. *Journal of the American Geriatrics Society*.

[19] Kundu S (2021). AI in medicine must be explainable. *Nature Medicine*.

[20] Cutillo CM, Sharma KR, Foschini L, Kundu S, Mackintosh M, Mandl KD (2020). Machine intelligence in healthcare-perspectives on trustworthiness, explainability, usability, and transparency. *NPJ Digital Medicine*.

[21] Wang F, Kaushal R, Khullar D (2020). Should Health Care Demand Interpretable Artificial Intelligence or Accept “Black Box” Medicine. *Annals of Internal Medicine*.

[22] Mutasa S, Sun S, Ha R (2020). Understanding artificial intelligence based radiology studies: What is overfitting. *Clinical Imaging*.

[23] Hosseini M, Powell M, Collins J, Callahan-Flintoft C, Jones W, Bowman H (2020). I tried a bunch of things: The dangers of unexpected overfitting in classification of brain data. *Neuroscience and Biobehavioral Reviews*.

[24] Nordin N, Zainol Z, Mohd Noor MH, Chan LF (2023). An explainable predictive model for suicide attempt risk using an ensemble learning and Shapley Additive Explanations (SHAP) approach. *Asian Journal of Psychiatry*.

[25] Shang H, Chu Q, Ji M, Guo J, Ye H, Zheng S (2022). A retrospective study of mortality for perioperative cardiac arrests toward a personalized treatment. *Scientific Reports*.

[26] Harford S, Darabi H, Heinert S, Weber J, Campbell T, Kotini-Shah P (2022). Utilizing community level factors to improve prediction of out of hospital cardiac arrest outcome using machine learning. *Resuscitation*.

[27] Deng Y, Cheng S, Huang H, Liu X, Yu Y, Gu M (2022). Toward Better Risk Stratification for Implantable Cardioverter-Defibrillator Recipients: Implications of Explainable Machine Learning Models. *Journal of Cardiovascular Development and Disease*.

[28] Debjit K, Islam MS, Rahman MA, Pinki FT, Nath RD, Al-Ahmadi S (2022). An Improved Machine-Learning Approach for COVID-19 Prediction Using Harris Hawks Optimization and Feature Analysis Using SHAP. *Diagnostics*.

[29] Wang K, Tian J, Zheng C, Yang H, Ren J, Liu Y (2021). Interpretable prediction of 3-year all-cause mortality in patients with heart failure caused by coronary heart disease based on machine learning and SHAP. *Computers in Biology and Medicine*.

[30] Wong XY, Ang YK, Li K, Chin YH, Lam SSW, Tan KBK (2022). Development and validation of the SARICA score to predict survival after return of spontaneous circulation in out of hospital cardiac arrest using an interpretable machine learning framework. *Resuscitation*.

[31] Martínez-Sellés M, Marina-Breysse M (2023). Current and Future Use of Artificial Intelligence in Electrocardiography. *Journal of Cardiovascular Development and Disease*.

[32] Sammani A, van de Leur RR, Henkens MTHM, Meine M, Loh P, Hassink RJ (2022). Life-threatening ventricular arrhythmia prediction in patients with dilated cardiomyopathy using explainable electrocardiogram-based deep neural networks. *Europace: European Pacing, Arrhythmias, and Cardiac Electrophysiology*.

[33] Chen X, Chen H, Nan S, Kong X, Duan H, Zhu H (2023). Dealing With Missing, Imbalanced, and Sparse Features During the Development of a Prediction Model for Sudden Death Using Emergency Medicine Data: Machine Learning Approach. *JMIR Medical Informatics*.

[34] Trayanova NA, Topol EJ (2022). Deep learning a person’s risk of sudden cardiac death. *The Lancet*.

[35] Barker J, Li X, Khavandi S, Koeckerling D, Mavilakandy A, Pepper C (2022). Machine learning in sudden cardiac death risk prediction: a systematic review. *Europace: European Pacing, Arrhythmias, and Cardiac Electrophysiology*.

