# Supplementary material for: Clinical Validation of Explainable Deep Learning Model for Predicting the Mortality of In-Hospital Cardiac Arrest Using Diagnosis Codes of Electronic Health Records
Source: Rev Cardiovasc Med. 2023 Sep 21;24(9):265. doi: 10.31083/j.rcm2409265 (PMC11270098; doi:10.31083/j.rcm2409265)
Supplement: Supplementary file 1 [file 2153-8174-24-9-265-s1.zip › 2153-8174-24-9-265-s1.docx]

**Supplementary materials**

Supplementary information: Deep SHapley Additive exPlanations (D-SHAP) Deep Learning Models

The intuition for D-SHAP comes from the Taylor series formulation:

$$f\left( z \right)=f\left( z^{0} \right)+f_{z}^{'}*\left( z-z^{0} \right)+\Delta\left( z-z^{0} \right)\text{ }\text{ }\text{(}\text{1)}$$

that represents any function $f(z)$ as a linear function using the reference $z^{0}$ plus the error term $\Delta\left( z-z^{0} \right)$. If the $\Delta$ term is small enough, a linear transformation holds with respect to the local input and output where the coefficients $f_{z}^{'}$ can be analytically approximated by $\left( f\left( z \right)-f\left( z^{0} \right) \right)/\left( z-z^{0} \right)$ without actually using the derivative function. In D-SHAP, based on the above statement, we have the following: For any local output $f(z)$, a linear function $\phi\left( f,z \right)$ with respect to the local input $z= \left\{ z_{1},\ldots, z_{N} \right\} \in\mathbb{R}^{N}$ can be found to approximate it, with

$$\phi\left( f,z \right)= \sum_{i}^{N} m_{z_{i}}f*\left( z_{i}-E\left( z_{i} \right) \right)\approx f_{\left( z \right)}-f\left( E_{\left( z \right)} \right)\text{.}\text{ }\text{ (2)}$$

Here $E_{\left( z_{i} \right)}$ denotes the expected value of each element in the local input. The term $\left( z_{i}-E\left( z_{i} \right) \right)$ indicates the amount of deviation of the element $z_{i}$ of a local input from the reference, the latter being given by the average state of the function $f$. In practice, the local expected value $E\left( z_{i} \right)$ can be approximated by the empirical mean value $\bar{z_{i}}$. The scalar $m_{z_{i}}f$, which is the slope between $\left( z_{i},f_{\left( z \right)} \right)$ and $\left( E_{\left( z_{i} \right)},f\left( E\left( z \right) \right) \right)$, is called the SHAP value of the function $f$ corresponding to the $i\text{th}$ feature value $z_{i}$. Meanwhile, for a differentiable neural network $F_{\left( x \right)}=f_{3}\left( f_{1}\left( x \right),f_{2}\left( x \right) \right)$ (see Supplementary Fig. 1 as an example), the D-SHAP value of $x$ can be calculated by applying the chain rule:

$\forall z\in\left\{ x_{1},x_{2},x \right\}\text{ } \text{a}\text{n}\text{d } \forall g\in\left\{ f_{1,} f_{2,} f_{3} \right\}\text{ } m_{zg}=\frac{\Phi\left( g,z \right)}{z-E\left( z \right)} \text{ } \text{from Equation (2)}$,

$$m_{xf_{3}}=\sum_{j=1}^{2} m_{xf_{j}}m_{x_{j}f_{3}}\text{ }\text{(chain rule).}\text{ }\text{ (3)}$$

With the method above, the D-SHAP value of any differentiable neural network can be calculated using the current neural network framework by replacing the derivatives with the SHAP value in each layer.

Supplementary Fig. 1. Derivatives of SHAP values for neural networks using back-propagation [1].


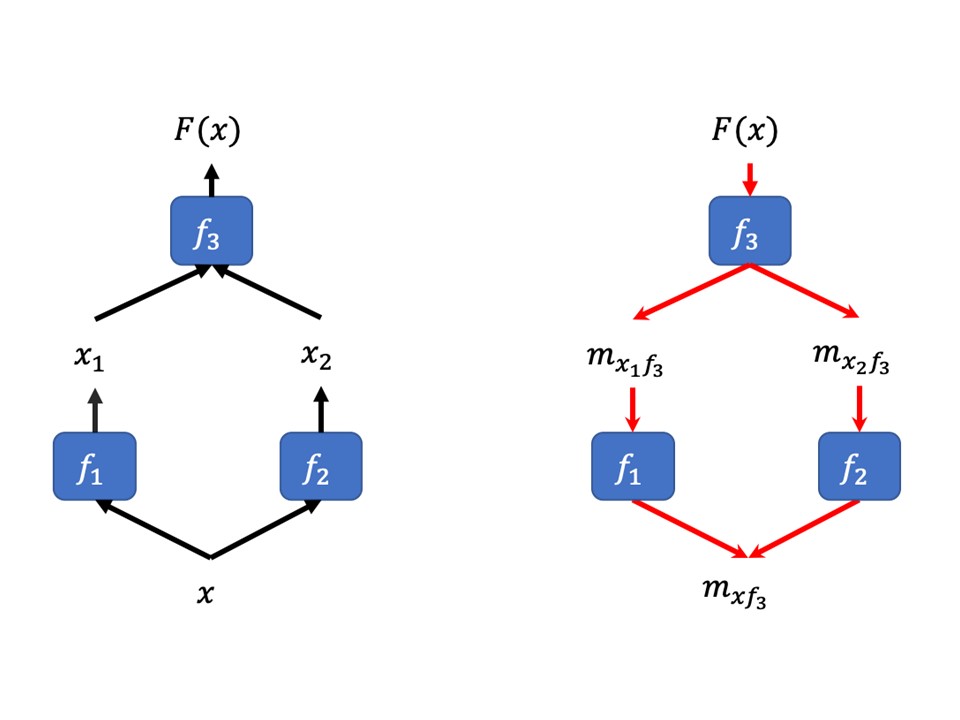


With the D-SHAP framework, we can effectively calculate the SHAP value for each input dimension. The input vector has 707 dimensions as was demonstrated in a previous report [2]. Accordingly, the SHAP value of the input is a 707-dimensional vector, within which a 128-dimensional vector can be found as the SHAP value for the diagnosis code.

From Equation (2) and the linearity assumption, it is straightforward to investigate the impact of a single dimension on the output with the SHAP value since the single dimension of the input is generally intuitive to a human. However, it is difficult to determine the impact of the SHAP values for either latent or vector inputs as changes in the input are not intuitive (see Supplementary Table 1 as an example). In our case, the diagnosis code is a latent vector of 128 dimensions, and accordingly the SHAP value of the diagnosis code is also a 128-dimensional vector. It is tedious and meaningless to interpret the 128-dimensional SHAP value dimension by dimension. In this work, several aggregation methods are used to compress the 128-dimensional SHAP value for the diagnosis code to a scalar that can

- preserve the information of the original SHAP value;
- produce an interpretation result consistent with the human expert.

Supplementary Table 1. Interpreting SHAP value for scalar and vector inputs. The three-dimensional diagnosis vector is used as a vector input example.

|  | Age | Diagnosis Code |
| --- | --- | --- |
| Input value | 30 | [1,0,2] |
| SHAP value | 0.2 | [3,2,−1] |
| Interpretation | Increase the age by 1 would increase the output by 0.2 | Increase the 1st/2nd/3rd dimension of  the latent vector would increase the output by 3/2/-1 |

Interpretation of the SHAP value could explain the change in output corresponding to a change in the input. However, the impact of the feature on the output is more important for clinical researchers and was prioritized in this work. From Equation (2), $m_{z_{i}f}*\left( z_{i}-E\left( z_{i} \right) \right)$ is a scalar that describes the offset of the output caused by the input dimension $z_{i}$. The impact of a latent vector can be easily captured by summing up all dimensions of the input offset.

Intuitively, the patient’s historical diagnosis, especially for certain chronic diseases, could have an impact on current mortality prediction. The historical diagnosis SHAP value is used to describe the overall impact of previous diagnosis codes on the current prediction. Specifically, the average of all historical diagnosis SHAP values is used to represent their overall impact in comparison with the current diagnosis SHAP value. Given an input $Z=\left[ Z_{0},\ldots,Z_{n} \right]\in\mathbb{R}^{N}$, we have the vector feature $Z_{ij}=\left[ Z_{i},\ldots,Z_{i} \right]$ with $0 \leq i<j\leq n$ and the SHAP value $\left[ m_{z_{i}f},\ldots,m_{Z_{i}f} \right]$ corresponding to $Z_{ij}$. The SHAP impact of feature $z_{ij}$, $\phi_{linear}\left( f,z_{ij} \right)$, can be calculated from Equation (2):

$$\phi_{linear}\left( f,z_{ij} \right)=\sum_{k=i}^{j} m_{z_{k}}f*\left( z_{k}-E\left( z_{k} \right) \right)\text{.}\text{ }\text{ (4)}$$

With Equation (4), a simple linear combination can be used to evaluate the impact for vector features.

**Reference**

[1] Lundberg S, Lee SI. A Unified Approach to Interpreting Model Predictions. arXiv*.* 2017.

[2] Chi CY, Ao S, Winkler A, Fu KC, Xu J, Ho YL*, et al.* Predicting the Mortality and Readmission of In-Hospital Cardiac Arrest Patients With Electronic Health Records: A Machine Learning Approach. Journal of Medical Internet Research*.* 2021; 23.
